# CS-MRI Reconstruction Using an Improved GAN with Dilated Residual Networks and Channel Attention Mechanism

**DOI:** 10.3390/s23187685

**Published:** 2023-09-06

**Authors:** Xia Li, Hui Zhang, Hao Yang, Tie-Qiang Li

**Affiliations:** 1 College of Information Engineering, China Jiliang University, Hangzhou 310018, China; 2 Department of Clinical Science, Intervention, and Technology, Karolinska Institute, 14186 Stockholm, Sweden; 3 Department of Medical Radiation and Nuclear Medicine, Karolinska University Hospital, 17176 Stockholm, Sweden

**Keywords:** compressed sensing MRI, GAN, U-net, dilated residual blocks, channel attention mechanism

## Abstract

Compressed sensing (CS) MRI has shown great potential in enhancing time efficiency. Deep learning techniques, specifically generative adversarial networks (GANs), have emerged as potent tools for speedy CS-MRI reconstruction. Yet, as the complexity of deep learning reconstruction models increases, this can lead to prolonged reconstruction time and challenges in achieving convergence. In this study, we present a novel GAN-based model that delivers superior performance without the model complexity escalating. Our generator module, built on the U-net architecture, incorporates dilated residual (DR) networks, thus expanding the network’s receptive field without increasing parameters or computational load. At every step of the downsampling path, this revamped generator module includes a DR network, with the dilation rates adjusted according to the depth of the network layer. Moreover, we have introduced a channel attention mechanism (CAM) to distinguish between channels and reduce background noise, thereby focusing on key information. This mechanism adeptly combines global maximum and average pooling approaches to refine channel attention. We conducted comprehensive experiments with the designed model using public domain MRI datasets of the human brain. Ablation studies affirmed the efficacy of the modified modules within the network. Incorporating DR networks and CAM elevated the peak signal-to-noise ratios (PSNR) of the reconstructed images by about 1.2 and 0.8 dB, respectively, on average, even at 10× CS acceleration. Compared to other relevant models, our proposed model exhibits exceptional performance, achieving not only excellent stability but also outperforming most of the compared networks in terms of PSNR and SSIM. When compared with U-net, DR-CAM-GAN’s average gains in SSIM and PSNR were 14% and 15%, respectively. Its MSE was reduced by a factor that ranged from two to seven. The model presents a promising pathway for enhancing the efficiency and quality of CS-MRI reconstruction.

## 1. Introduction

Compressed sensing (CS) is a promising technique that capitalizes on the sparsity property for signal recovery [1]. CS-based methods have been increasingly employed to enhance MRI time efficiency. Introduced by Donoho in 2006 [2], the CS method, also known as compressed or sparse sampling, was first applied to fast MRI by Lustig et al. [3]. CS-MRI leverages sparse sampling and convex optimization algorithms to improve clinical MRI time efficiency [4]. Although CS-MRI surpasses the Nyquist–Shannon sampling barrier, its acceleration rate remains limited. Current sparse transforms in CS-MRI struggle to capture intricate image details [5], and iterative calculations in the nonlinear optimization solver prolong the reconstruction time [6]. Inappropriate hyperparameter prediction may result in overly smooth, unnatural images [7]. However, the widespread influence of artificial intelligence (AI) and deep learning (DL) advancements [8] have enhanced various aspects of medical image reconstruction, including their speed, accuracy, and robustness [9], making them increasingly important for fast CS-MRI reconstruction.

The use of deep learning techniques, such as generative adversarial networks (GANs) [10,11], has emerged as a leading approach to CS-MRI reconstruction. These techniques have enabled high-quality reconstruction without increasing model complexity. Convolutional neural networks (CNNs) are some of the most prominent DL models used for CS-MRI reconstruction. They excel in learning the non-linear mapping between undersampled and fully sampled MRI images. Models like U-net and its variants [12] have been particularly successful due to their ability to extract and combine features at different levels. Recurrent neural networks (RNNs) and specifically their long short-term memory (LSTM) variant have also been used in CS-MRI reconstruction [13]. They take advantage of the temporal correlation in dynamic MRI data, thus improving the reconstruction quality of dynamic sequences. The GAN-based models have also shown potential in CS-MRI reconstruction [11,14,15,16,17]. The competitive learning process between the generator and discriminator networks results in images with high perceptual quality. Some of the mainstream GAN-based CS-MRI reconstruction models are summarized below: the CycleGAN [18] model has been used for unsupervised image translation, where the goal is to transform images from one domain to another. In CS-MRI, CycleGAN can be used to map undersampled k-space data to fully sampled k-space data. The inherent cycle consistency loss helps to preserve the important structures in the MRI images, leading to better reconstruction. The pix2pix GAN model [19], also known as the image-to-image translation GAN, has been used in CS-MRI for mapping the undersampled MRI images to their fully sampled counterparts. This model has shown promising results in generating high-quality reconstructions, although the quality of the output largely depends on the quality of the paired data. The deep convolutional GAN (DCGAN) model [20] has been used in CS-MRI to learn the mapping between the undersampled and fully sampled MRI data. The deep convolutional layers in this model can better capture the complex patterns in the MRI data, thereby producing high-quality reconstructed images. The Wasserstein GAN (WGAN) model [21] uses the Wasserstein loss instead of the traditional GAN loss to stabilize the training process and improve the quality of the generated images. WGAN has been used in CS-MRI to generate more realistic reconstructions and better preserve the details of the original images. The progressive GAN model [22] adopts a new training methodology that grows both the generator and discriminator progressively, adding layers that model increasingly fine details as the training progresses. This method allows the model to effectively generate high-resolution details, leading to improved CS-MRI reconstruction. In the last three years, of deep-learning-based novel algorithms for CS-MRI reconstruction have continued to emerge [23,24,25,26,27,28]. For example, the projection-based cascaded U-Net model [23]; the geometric distillation network, used to unfold the model-based CS-MRI optimization problem [24]; the iterative fusion model, used to integrate the image- and gradient-based priors into reconstruction [25]; the interpretable network, which has two-grid cycle and geometric prior distillation [26]; and the fast iterative shrinkage thresholding network, used for high throughput reconstruction [27]. The new GAN-powered algorithms have also continued to grow [29,30,31,32], including ESSGAN [29], DBGAN [30], CoVeGAN [31], and SEPGAN [32]. In ESSGAN [29], structurally strengthened connections were introduced to enhance feature propagation and reuse the in-between concatenated convolutional autoencoders in addition to residual blocks. In DBGAN [30], the dual-branch GAN model uses cross-stage skip connection between two end-to-end-cascaded U-Nets to widen the channels for feature propagation in the generator. In the complex-valued GAN (Co-VeGAN) network [31], the use of complex-valued weights and operations was explored in addition to the use of a complex-valued activation function that is sensitive to the input phase. In SepGAN [32], depth-wise separable convolution was utilized as the basic component to reduce the number of learning parameters. Moreover, attention-enhanced GAN networks [33,34,35] have become more and more powerful [36]. Besides spatial [34] and channel attention mechanisms [33], transformer-based GAN networks have become quite effective [35]. The development of GAN-powered frameworks for CS-MRI reconstruction has been comprehensively summarized in recent reviews [36,37,38].

Deep-learning-based methods have shown impressive results in image and signal reconstruction. They often outperform conventional algorithms in terms of both speed and quality [39]. However, many models require increased network depth and width, which complicates training and prolongs the reconstruction time [21,40]. In this study, we improved upon a GAN-based model for the construction of CS-MRI. The generator module of the model was derived from the U-Net architectureby integrating dilated residual (DR) structures to expand the network’s receptive field, while avoiding an increase in parameters or computation. At each step of the downsampling path, the revamped generator module incorporates three such structures with varying dilation rates depending on the depth of the network layer [41,42]. To concentrate on essential information, we also introduced a channel attention mechanism (CAM) that distinguishes between channels and reduces background noise [43]. This mechanism integrates global maximum and average pooling for more precise channel attention. We’ve assigned this GAN-based CS-MRI reconstruction model, equipped with DR networks and CAM, the name DR-CAM-GAN. 

Using public domain MRI datasets for the human brain, we conducted extensive reconstruction experiments with the designed DR-CAM-GAN model at different undersampling levels. To assess the performance of the model, we compared the reconstructed image qualities with the results of a few reference models, including CRNN [13], DAGAN [15] and RefineGAN [44]. The image quality was evaluated according to the peak signal-to-noise ratio (PSNR), structural similarity (SSIM), and mean square error (MSE) [45]. The features of the study we would like to highlight are as follows: (1) Dilated residual networks with varying dilation rates to fully satisfy the receptive field [46], a channel attention mechanism for refining network resource allocation [47], and a multi-scale information fusion module for feature fusion and improved MRI reconstruction quality [48]. (2) A discriminator structure design that avoids max pooling layers and implements feature downsampling through varied convolution strides, with batch normalization to address gradient vanishing. (3) A combination of four loss functions—pixel-wise image-domain mean square error loss, frequency-domain MSE loss, perceptual VGG loss, and adversarial loss—assigned different weights to enhance reconstruction quality and visual perception.

## 2. Materials and Methods

### 2.1. Datasets and Data Processing 

To demonstrate the effectiveness of the proposed model, we utilized publicly available T_1_-weighted magnetization-prepared rapid gradient echo (MPRAGE) datasets of the human brain for testing, including the diencephalon challenge dataset of the MICCAI 2013 grand challenge (https://www.synapse.org/, accessed on 20 May 2022) and the Open Access Series of Imaging Studies (OASIS) dataset (https://www.oasis-brains.org/, accessed on 20 May 2022), which contains neuroimaging data for 1378 participants collected over a 30-year period. There are 176 image slices in each 3D T_1_-weighted MPRAGE file. We randomly shuffled these files, allocating 70% for training, 10% for validation, and 20% for testing. The training set updates the network parameters according to the gradient descent approach to best fit the real data distribution, while the validation set helps with hyperparameter fine-tuning, identifying overfitting, and selecting more accurate models. 

We utilized a fast Fourier transform procedure to convert the images into fully sampled k-space data within the complex domain. To generate undersampled images for CS-MRI reconstruction at different sampling rates, we extracted the k-space data at sub-Nyquist rates of 10%, 20%, 30%, and 50%, then conducted zero-filling on the undersampled k-space data and applied an inverse Fourier transform. This process was intended to demonstrate the adaptability of our proposed model to various CS-MRI sampling rates. During the training of the reconstruction model, the subsampled k-space data were used as input to generate output images that matched the Fourier transforms of fully sampled k-space data. Sparsity plays a crucial role in CS-MRI, particularly in the wavelet or Fourier domain. It is essential for the CS-MRI data sampling pattern to be incoherent with the sparse basis. Typically, variable-density random undersampling in the k-space is employed to achieve this. Since the central k-space region contains important high-energy information for image quality and tissue contrast, and higher k-space data contribute to image details and fine structures [12], we always included the central 4% low-frequency information to ensure good image quality during undersampling. To simulate incoherent k-space data undersampling, we adopted a Monte Carlo’s random undersampling strategy based on Gaussian sampling distribution. This approach exploits the high energy at the center of the k-space while also including some sparse-distributed high-frequency k-space data points needed to preserve fine structural details and correct for aliasing artifacts.

Given the importance of extensive datasets for deep learning models and the limited availability of medical images, we utilized data augmentation techniques to expand our datasets and bolster model resilience. To achieve this, we implemented online stochastic augmentation, generating random augmentations for a data batch [49], which not only enhanced model stability but also alleviated storage constraints as compared to offline augmentation methods. We employed four different random enhancement methods with equal probability to ensure low data repeatability. We divided the data into four equal parts, augmenting each by flipping up and down, translating, mirroring horizontally, or rotating by 90 degrees. These methods reduce model sensitivity to target position, accelerate model convergence, and improve performance.

### 2.2. The Proposed Reconstruction Model

**Figure 1:**
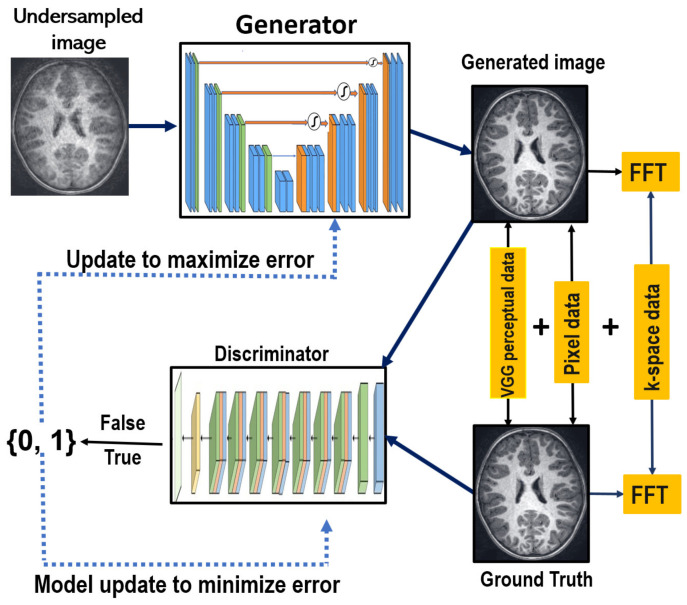
Schematic illustration of the improved GAN model with dilated residual networks and a channel attention mechanism (DR-CAM-GAN), intended for use in CS-MRI reconstruction.

GAN-based models are innovative deep generative models that leverage game theory and competitive learning between a generator and a discriminator to enhance the network’s fitting ability. The proposed DR-CAM-GAN framework consists of generator and discriminator modules. The generator module takes undersampled MR images and outputs reconstructed images after multiple levels of convolutional operations, while the discriminator classifies de-aliased reconstructed images from fully sampled ground truth images. The outline of the proposed GAN framework is displayed in Figure 1.

**Figure 2:**
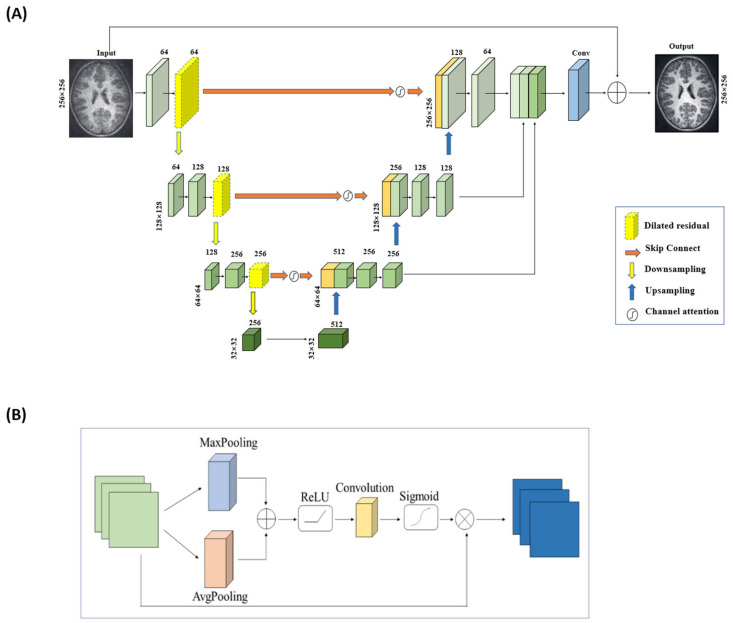
The U-net-based generator architecture (**A**) with integrated DR blocks and CAM (**B**).

In the DR-CAM-GAN framework, we use a U-net base structure for the generator, with an encoding layer for feature extraction and a decoding layer for feature amplification at each stage. Skip connections transfer information from the encoding layer to the corresponding decoding layer, enabling feature fusion and providing more accurate detailed features for reconstruction. To alleviate gradient disappearance or network degradation as the network depth increases, we modified the residual structures by replacing the second standard convolution with a dilated convolution. This expands the network’s receptive field without increasing parameters or computation. Dilated convolution is a technique that expands the kernel size by inserting holes between the kernel elements. In other words, the computation is the same as ordinary convolution, but it involves pixel skipping, so that the kernel can cover a larger area of the input feature map. In a regular convolution operation, a filter of a fixed size slides over the input feature map, and the values in the filter are multiplied with the corresponding values in the input feature map to produce a single output value. The receptive field of a neuron in the output feature map is defined as the area in the input feature map that the filter can cover. Therefore, the size of the receptive field is determined by the size of the filter and the stride of the convolution. In a dilated convolution operation, the filter is “dilated” by inserting space between the kernel elements, and the gaps are determined by an adjustable hyperparameter called the dilation rate, which effectively increases the receptive field of the filter without increasing the number of parameters. This can be useful in situations where a larger receptive field is needed, but the size of the filter is limited. Our generator structure employs three dilated residual blocks with different dilation rates (1, 2, and 3), which vary depending on the network layer’s depth. We used skip connections in the U-net structure [50] to fuse deep and shallow features, improving MR image reconstruction quality. To better focus on key information, we propose a new channel attention mechanism that establishes dependencies between channels, suppressing background information. This mechanism combines global maximum pooling and global average pooling to obtain more accurate channel attention. Despite the dilated residual structure and channel attention mechanism’s strengths, the decoding layer structure is a bottom-up process, which can lead to information loss or corruption. We employed a multi-scale information fusion module to aggregate features from multiple levels, using linear interpolation techniques for upsampling to avoid midway detail information loss and fully utilize the information. Figure 2 depicts the generator with integrated DR blocks and CAM.

The discriminator’s primary function is to determine the input MR images’ source. It consists of a series of convolutions, employing batch normalization after each convolution to normalize input and avoid gradient disappearance. The discriminator structure follows Radford et al.’s [20] architectural guidelines, using convolution stride variations for feature downsampling instead of max pooling layers. We utilize LeakyReLU for enhanced nonlinearity and a sigmoid function to determine whether an MR image is fully sampled or reconstructed. 

### 2.3. The Loss Function

The GAN model is trained in an alternating fashion: the discriminator trains for one or more epochs, then the generator is trained. This procedure is repeated until the targeted loss or the ultimate number of iterations is reached. To enhance the reconstruction quality and visual perception of our model, we used a combination of pixel-wise image-domain mean square error (MSE) loss, frequency-domain MSE loss, perceptual VGG loss, and adversarial loss [51], assigning them different weights to form the generator’s loss function. The combined loss function for the generator is given by:

(1) $$L_{combine}=\alpha L_{iMSE}+\beta L_{fMSE}+\delta L_{VGG}+L_{GEN}$$

where the hyperparameters α,β,δ are the weights associated with different loss terms. Balancing these weights is achieved by setting them for different undersampling ratios. We trained the network with α=15, β=0.1, and δ=0.0025, as used previously in GAN-related model training. Pixel loss ensures positive similarity between the reconstructed and original images by calculating the MSE between each pixel point of the reconstructed MRI and the fully sampled image:

(2) $$L_{iMSE}=\frac{1}{2}{\left\|X_{t}-X_{u}\right\|}_{2}^{2}$$

where $X_{t}$ represents the fully sampled image and $X_{u}$ is the reconstructed image generated by a cascaded convolutional neural network. Frequency-domain MSE loss enhances the similarity between a fully sampled image and a reconstructed image, defined as:

(3) $$L_{fMSE}=\frac{1}{2}{\left\|Y_{t}-Y_{u}\right\|}_{2}^{2}$$

where $Y_{t}$ and $Y_{u}$ are the corresponding frequency-domain data of $X_{t}$ and $X_{u}$. Pixel- and frequency-domain losses yield reconstructed MRIs with higher PSNR and lower MSE. However, optimizing MSE may result in the loss of high-frequency information, leading to over-smoothed images that affect human visual perception. To address this issue, we introduced perceptual loss from Gatys et al. [30] to reduce the gap between the reconstructed MRI and the fully sampled image in feature space, obtaining higher texture similarity:

(4) $$L_{VGG}=\frac{1}{2}{\left\|f_{VGG}(X_{t})-f_{VGG}(X_{u})\right\|}_{2}^{2}$$

where $f_{VGG}$ refers to the VGG16 network. 

The adversarial loss in the GAN network represents the difference between the predicted probability distribution produced by the discriminator and the actual probability distribution of real samples. It is expressed as the log of the discriminator probability distribution for the generated image data:

(5) $$L_{GEN}=-\log\left(D_{\theta_{d}}\left(G_{\theta_{g}}\left(X_{u}\right)\right)\right)$$

While the generator aims to produce a better image from the undersampled data by focusing on improving image quality, the discriminator’s primary objective is to maximize the probability assigned to real and fake images: that is, to distinguish the image produced by the generator from the fully sampled ground truth image. The two modules are engaged in a min-max game, where simultaneous improvements are achieved for both modules through competition. Mathematically, the model is seeking to minimize the average binary cross entropy. This can be expressed as follows [36]:

(6) $$\;L_{D}=-\log\left(D_{\theta_{d}}\left(X_{t}\right)\right)-\log\left(1-D_{\theta_{g}}\left(X_{u}\right)\right)\;$$

where $\log\left(D_{\theta_{d}}\left(X_{t}\right)\right)$ represents the log of the discriminator probability distribution for the fully sampled image data, and $\log\left(1-D_{\theta_{g}}\left(X_{u}\right)\right)$ is the log of the invert probability distribution for the generated images from the undersampled data. For the discriminator, minimizing the $L_{D}$ loss function is equivalent to maximizing the judgement accuracy of the fully sampled image and the network’s reconstructed image using undersampled data.

### 2.4. Model Training 

We employed an NVIDIA Geforce 3060 GPU for both the training and testing phases, utilizing the PyTorch development environment and a model with a total of 41.84 MB of parameters. Our model was trained using the ADAM optimizer with set parameters: β1 = 0.9, β2 = 0.999, an initial learning rate of 0.0001, a learning rate decay factor of 0.5, and an update interval of 10 epochs for the learning rate. To mitigate overfitting, we used MSE as the evaluation metric to optimize our model. Training was halted and the current model saved if the observed MSE was lower than any MSE from the subsequent 20 epochs. Each epoch, which included the computation of PSNR, SSIM, and MSE for the validation sets, was completed within approximately 20 min.

We conducted ablation studies to ascertain the contribution of individual elements to our proposed reconstruction model. By systematically altering the model, we gauged the performance of these variants in comparison to the original comprehensive model. We specifically scrutinized the effects of the dilated residual structure, channel attention mechanism, and multi-scale information fusion module on the model’s performance. This was accomplished by modifying or removing each component and evaluating the resultant model’s performance.

As detailed in Table 1, we compared the performance of five renowned CS-MRI reconstruction models—U-net [12], CRNN [13,52], DAGAN [15], RefineGAN [44], and ESSGAN [29]—using the same training datasets and similar training procedures as described above. We used three metrics (PSNR, SSIM, and MSE) to assess and compare the performance of the different models under different levels of undersampling. 

## 3. Results

**Figure 3:**
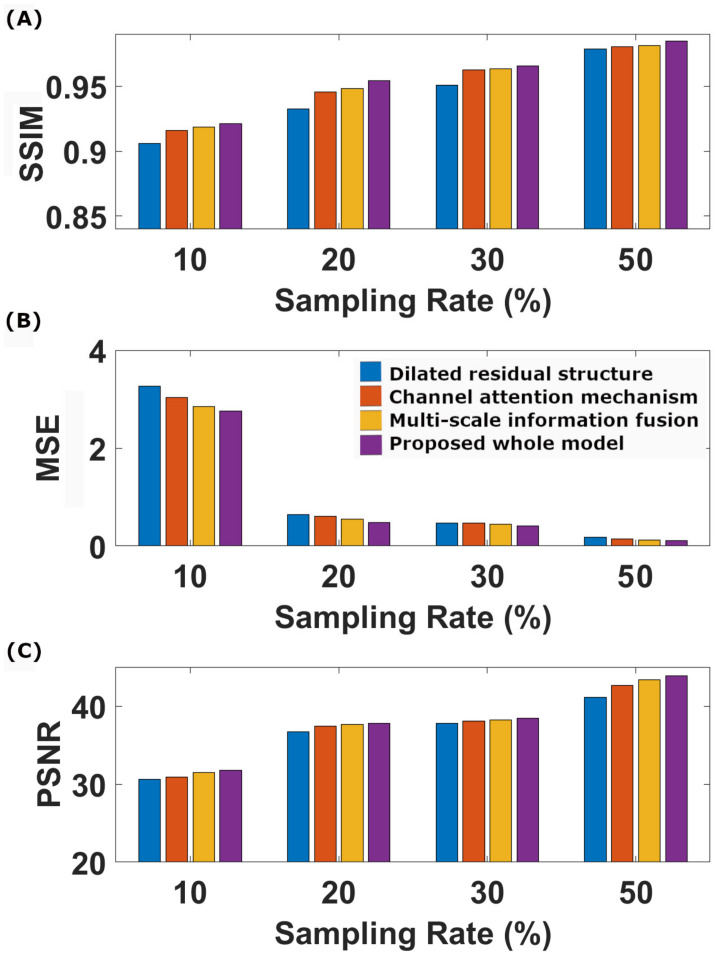
Bar graph of the SSIM (**A**), MSE (**B**), and PSNR (**C**) as a function of the CS undersampling rates for the different models.

Table 2 and Table 3 and Figure 3 present the quantitative outcomes of our ablation studies, encompassing evaluation metrics such as model size, training time, SSIM, MSE, and PSNR across four different undersampling levels. The full model we propose, DR-CAM-GAN, outperforms in all three metrics. The DR networks elevate PSNR, SSIM, and MSE as being 1.6–6.3%, 0.6–2.3%, and 14–64%, respectively, contingent on the undersampling levels. Introducing the CAM results in improvements of 0.9–3.0% for PSNR, 0.4–0.9 for SSIM, and 10–26% for MSE. Multi-scale information fusion modestly boosts PSNR, SSIM, and MSE by 0.4–1.3%, 0.3–0.6, and 3–15%, respectively. Among these different innovative modifications, the DR networks contribute most significantly to performance enhancement, followed by the improvement provided by the CAM.

**Figure 4:**
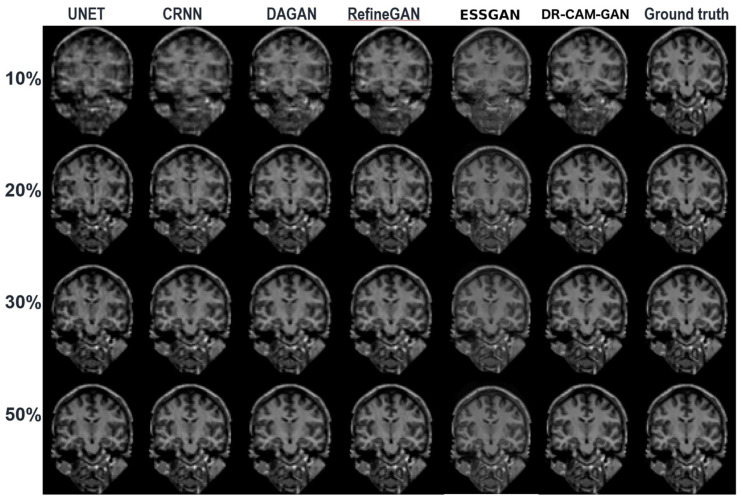
A coronal slice reconstructed from a T1-weighted MPRAGE volume by five distinct DL models (column-wise) at four different CS undersampling rates (row-wise).

**Figure 5:**
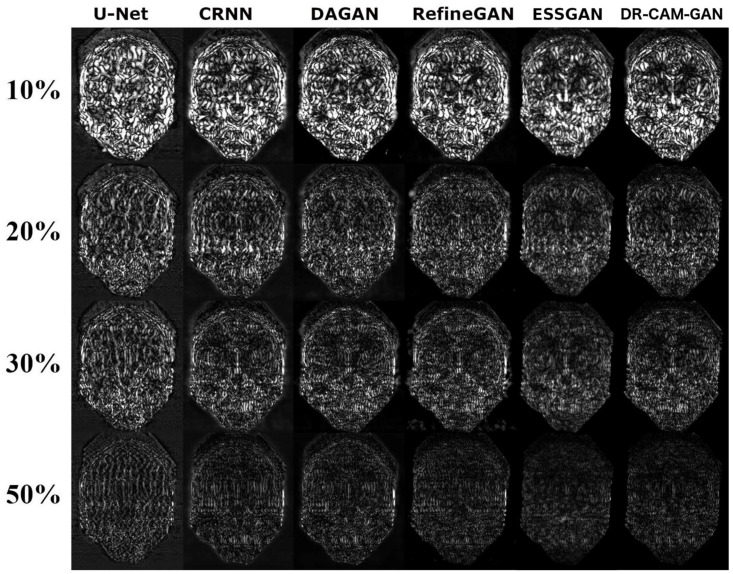
The MSE maps corresponding to the coronal slice depicted in Figure 4, illustrating the results from six DL models (column-wise) at four different CS undersampling rates (row-wise).

Figure 4 depicts a representative coronal slice of images reconstructed by various models, including our proposed DR-CAM-GAN framework, at four distinct undersampling levels. Figure 5 demonstrates the MSE outcomes for the same slice. At lower sampling rates (10% and 20%), models such as U-net, CRNN, and DAGAN struggle with signal recovery, leading to blurring in the reconstructed images. In stark contrast, both the RefineGAN and proposed DR-CAM-GAN models display superior performance, with less blur and enhanced detail recovery in brain structure. The DR-CAM-GAN framework excels, even surpassing the highly effective RefineGAN model. At a 20% sampling rate, the network largely succeeds in reconstructing the brain structure, although edge details remain slightly blurred with minor visible artifacts. On the other hand, reconstructions from the CRNN and DAGAN models appear noisy and present a more significant loss of brain anatomy details.

**Figure 6:**
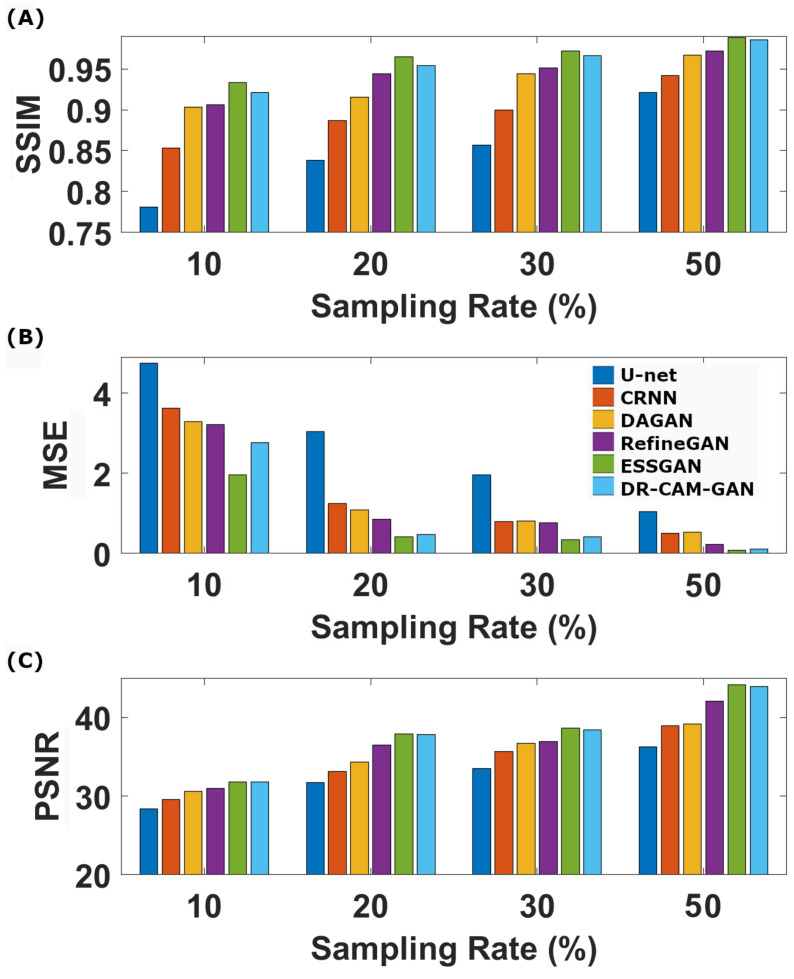
The SSIM (**A**), MSE (**B**), and PSNR (**C**) as a function of the CS undersampling rates for the different DL models.

Table 4 and Figure 6 encapsulate the quantitative evaluations of the reconstructed image quality. DL models showcase robust performance, even at elevated undersampling rates, with the various quality metrics (PSNR, SSIM, and MSE) following a similar trajectory. Our proposed DR-CAM-GAN model stands out, demonstrating remarkable performance and stability. Regardless of the sampling rate, DR-CAM-GAN consistently outperforms most of the compared literature models, except for the more recent ESSGAN model. Compared with the ESSGAN, DR-CAM-GAN’s performances in PSNR and SSIM are systematically lower; however, the differences are quite marginal (0.3 vs. 1.3% in SSIM and 0.07 vs. 0.22 dB in PSNR). When pitted against U-net, DR-CAM-GAN’s gains in SSIM and PSNR range from 6 to 15% and from 11 to 17% respectively. Even compared to RefineGAN, improvements in SSIM and PSNR consistently surpass 1 and 3%, respectively, depending on the undersampling levels. The reduction in its MSE is even more noteworthy, ranging from two to nine times lower, which is particularly impressive at higher sampling rates. 

## 4. Discussion

To summarize, our proposed DR-CAM-GAN model exhibits superior performance in image reconstruction compared to other DL models, even at elevated undersampling rates. Quantitative assessments indicated substantial improvements in PSNR, SSIM, and MSE metrics. The DR networks contribute the most significantly to this performance enhancement. The DR-CAM-GAN model consistently outperforms U-net, CRNN, DAGAN, and even the efficient RefineGAN, with notable improvements in image quality and noise reduction. Compared with the ESSGAN, DR-CAM-GAN’s performances in PSNR and SSIM are slightly lower. Overall, the DR-CAM-GAN model demonstrates good performance and stability and can effectively recover detailed brain structures from undersampled data.

The ablation study results presented in Table 2 and Table 3 and Figure 3 offer valuable insights into the contributions of each component of our proposed DR-CAM-GAN model. By evaluating the model’s performance using SSIM, MSE, and PSNR metrics across four distinct undersampling levels, we can better understand how each element impacts the overall performance. In summary, the ablation study highlights the importance of each component in the DR-CAM-GAN model. The DR networks are the primary driver of performance improvement, while the CAM mechanism and multi-scale information fusion provide valuable support. These combined elements allow the DR-CAM-GAN model to achieve superior performance across all evaluation metrics, demonstrating its effectiveness in reconstructing high-quality images from undersampled data. The DR networks play a crucial role in enhancing the model’s performance. The DR networks demonstrate their effectiveness in capturing multi-scale information and preserving the spatial structure of the images. This leads to better image quality and improved reconstruction, particularly in capturing fine details and maintaining the overall structure of the brain. The CAM mechanism, though it provides only a moderate contribution, is still an essential component of the model. It focuses on important features by adaptively weighing channel-wise information, thereby improving the model’s ability to recover specific details and suppress less relevant information. This results in better image quality and reduced noise. The multiscale information fusion further refines the model’s performance by moderately increasing PSNR, SSIM, and MSE. This component enables the model to incorporate information from different scales, ensuring that both global and local features are well-represented in the final reconstruction. This fusion process contributes to a more accurate and detailed image representation.

As suggested by the results of the ablation study, the superior performance can be attributed to the combination of the DR networks, CAM mechanism, and multi-scale information fusion. This combination allows the DR-CAM-GAN model to outperform most of the compared DL models and achieve exceptional performance and stability in CS image reconstruction tasks. The model comparison results showcased in Figure 4, Figure 5 and Figure 6 provide a comprehensive understanding of how the proposed DR-CAM-GAN model performs against other deep learning models in image reconstruction tasks. By examining the structure and reconstruction quality of various models, such as U-net, CRNN, DAGAN, RefineGAN, ESSGAN, and our proposed DR-CAM-GAN, we can evaluate their strengths and weaknesses and identify the factors contributing to the superior performance of the DR-CAM-GAN model.

The CRNN [52] is not a GAN-based architecture but a convolutional recurrent neural network embedded with the structure of traditional iterative algorithms. CRNN can efficiently model the recurrence of the iterative reconstruction stages. The U-Net [12] follows an encoder–decoder cascade architecture, where the encoder gradually compresses information into a lower-dimensional representation. Then the decoder decodes this information back to the original image dimension. Owing to the overall U-shaped structure, the architecture is named U-Net and is the basic construction unit for many variants of GAN generator modules. DAGAN [15] is a deep learning architecture for fast, de-aliasing CS-MRI reconstruction. Its generator network is a U-Net architecture with skip connections. RefineGAN [44] employs chained U-nets to form deeper generator and discriminator networks and further enhance the reconstruction quality. ESSGAN [29] consists of a structurally strengthened generator with strengthened connections that enhance feature propagation between the concatenated and strengthened convolutional autoencoders, in addition to strengthening the residual in residual blocks. In DR-CAM-GAN, we derived the generator module from a U-net model by integrating dilated residual structures of different dilation rates according to the depth in the encoder to expand the network’s receptive field, in addition to introducing CAM mechanisms. Overall, the architectures for RefineGAN and ESSGAN are more complex, with larger models and more parameters, which leads to an increase in training time (see Table 1). Compared with DAGAN and RefineGAN, our proposed DR-CAM-GAN method can improve image quality while reducing training time.

At low sampling rates (10 and 20%), the limited amount of sampled data poses a challenge for most models. U-net, CRNN, and DAGAN struggle to recover lost signals, resulting in blurry reconstructed images with less detail. This limitation can be attributed to these models’ inability to capture multi-scale information effectively, which is crucial for preserving the spatial structure and finer details of the images.

In contrast, RefineGAN and our DR-CAM-GAN model demonstrate better performance in reconstructing images with less blur and more detailed brain structures. The DR-CAM-GAN model, in particular, leverages its dilated residual networks, which enable the model to capture multi-scale information and preserve the spatial structure more effectively. Additionally, the channel attention mechanism focuses on relevant features, improving the model’s ability to recover specific details and suppress less important information.

The DR-CAM-GAN model outperforms even the highly efficient RefineGAN model. At a 20% sampling rate, the network recovers much of the brain structure. However, edge information remains somewhat blurred, and minor artifacts are present. Conversely, reconstruction results from CRNN and DAGAN models are noisy, with some loss of brain anatomy details.

Quantitative assessments of image quality, including those using PSNR, SSIM, and MSE metrics, further confirm the outstanding performance of the DR-CAM-GAN model. As shown in Table 4 and Figure 6, deep learning models demonstrate good performance even at high undersampling rates, but the DR-CAM-GAN model consistently achieves significant improvements in PSNR and SSIM compared to other networks. This superior performance can be attributed to the model’s ability to effectively capture multi-scale information and focus on relevant features through the DR networks and the CAM mechanism.

## 5. Conclusions

In conclusion, the DR-CAM-GAN model outperforms other deep learning models in image reconstruction tasks, even at high undersampling rates. Its superior performance and stability in recovering detailed brain structures stem from the integration of dilated residual networks, a channel attention mechanism, and multi-scale information fusion. The ablation study highlights the importance of DR networks, with the CAM mechanism and multi-scale fusion providing valuable support. Consequently, the DR-CAM-GAN model excels across all evaluation metrics, proving its effectiveness in reconstructing high-quality images from undersampled data. The model surpasses deep learning models like U-net, CRNN, DAGAN, and even RefineGAN, with quantitative assessments confirming its outstanding performance. The DR-CAM-GAN model’s success lies in its ability to capture multi-scale information and focus on relevant features. Ultimately, this model presents a promising solution for reconstructing high-quality images from undersampled data, with significant potential for various medical imaging and computer vision applications.

## Figures and Tables

**Table 1:** Summary of the different DL models assessed according to their performance in CS-MRI reconstruction.

Model	Reference	Parameters	Model Size	Batch	Epoch	Training
U-net	[12]	2.04 M	5.12 MB	16	200	3.6 h
CRNN	[52]	0.32 M	1.13 MB	16	100	2.4 h
DAGAN	[15]	98.60 M	376.16 MB	16	60	10.6 h
RefineGAN	[44]	40.91 M	105.34 MB	16	50	18.4 h
ESSGAN	[29]	35.71 M	74.50 MB	16	50	21.3 h
DR-CAM-GAN		15.77 M	41.84 MB	16	50	14.2 h

**Table 2:** Summary of the DR-CAM-GAN component efficacy as tested by ablation studies.

Ablation Component	Parameters	Model Size	Batch	Epoch	Training
Dilated residual structure	14.99 M	38.87 MB	16	50	12.9 h
Channel attention mechanism	15.68 M	41.51 MB	16	50	13.5 h
Multi-scale information fusion	15.74 M	41.72 MB	16	50	14.1 h

**Table 3:** Results of the ablation experiments with the DR-CAM-GAN framework.

Sampling	Ablation Comparison	SSIM	MSE ($\times 10^{-3}$)	PSNR (dB)
10%	Dilated Residual structure	0.9059	3.27	30.62
	Channel attention mechanism	0.9157	3.04	30.91
	Multi-scale information fusion	0.9184	2.85	31.49
	DR-CAM-GAN full model	0.9213	2.76	31.76
20%	Dilated Residual structure	0.9325	0.64	36.68
	Channel attention mechanism	0.9456	0.61	37.45
	Multi-scale information fusion	0.9479	0.55	37.63
	DR-CAM-GAN full model	0.9541	0.48	37.79
30%	Dilated Residual structure	0.9508	0.47	37.82
	Channel attention mechanism	0.9622	0.47	38.05
	Multi-scale information fusion	0.9631	0.44	38.21
	DR-CAM-GAN full model	0.9656	0.41	38.43
50%	Dilated Residual structure	0.9786	0.18	41.13
	Channel attention mechanism	0.9804	0.15	42.62
	Multi-scale information fusion	0.9812	0.12	43.35
	DR-CAM-GAN full model	0.9847	0.11	43.90

**Table 4:** Performance comparison for six different DL models at four different undersampling rates.

Sampling	Model	SSIM	MSE ($\times 10^{-3}$)	PSNR (dB)
10%	U-net	0.781	4.75	28.34
	CRNN	0.853	3.63	29.53
	DAGAN	0.903	3.29	30.59
	RefineGAN	0.906	3.22	30.97
	ESSGAN	0.933	1.96	31.83
	DR-CAM-GAN	0.921	2.76	31.76
20%	U-net	0.838	3.04	31.75
	CRNN	0.887	1.25	33.16
	DAGAN	0.915	1.09	34.32
	RefineGAN	0.944	0.85	36.50
	ESSGAN	0.965	0.41	37.86
	DR-CAM-GAN	0.954	0.48	37.79
30%	U-net	0.857	1.96	33.54
	CRNN	0.900	0.79	35.67
	DAGAN	0.944	0.81	36.69
	RefineGAN	0.951	0.77	36.95
	ESSGAN	0.972	0.35	38.62
	DR-CAM-GAN	0.966	0.41	38.43
50%	U-net	0.921	1.05	36.28
	CRNN	0.942	0.50	38.93
	DAGAN	0.967	0.53	39.18
	RefineGAN	0.972	0.23	42.05
	ESSGAN	0.988	0.08	44.12
	DR-CAM-GAN	0.985	0.11	43.90

## Data Availability

The study was based on public domain data openly accessible.
